# Univariate/Multivariate Genome-Wide Association Scans Using Data from Families and Unrelated Samples

**DOI:** 10.1371/journal.pone.0006502

**Published:** 2009-08-04

**Authors:** Lei Zhang, Yu-Fang Pei, Jian Li, Christopher J. Papasian, Hong-Wen Deng

**Affiliations:** 1 The Key Laboratory of Biomedical Information Engineering, Ministry of Education, Institute of Molecular Genetics, School of Life Science and Technology, Xi'an Jiaotong University, Xi'an, People's Republic of China; 2 Center of System Biomedical Sciences, Shanghai University of Science and Technology, Shanghai, People's Republic of China; 3 School of Medicine, University of Missouri-Kansas City, Kansas City, Missouri, United States of America; University of Chicago, United States of America

## Abstract

As genome-wide association studies (GWAS) are becoming more popular, two approaches, among others, could be considered in order to improve statistical power for identifying genes contributing subtle to moderate effects to human diseases. The first approach is to increase sample size, which could be achieved by combining both unrelated and familial subjects together. The second approach is to jointly analyze multiple correlated traits. In this study, by extending generalized estimating equations (GEEs), we propose a simple approach for performing univariate or multivariate association tests for the combined data of unrelated subjects and nuclear families. In particular, we correct for population stratification by integrating principal component analysis and transmission disequilibrium test strategies. The proposed method allows for multiple siblings as well as missing parental information. Simulation studies show that the proposed test has improved power compared to two popular methods, EIGENSTRAT and FBAT, by analyzing the combined data, while correcting for population stratification. In addition, joint analysis of bivariate traits has improved power over univariate analysis when pleiotropic effects are present. Application to the Genetic Analysis Workshop 16 (GAW16) data sets attests to the feasibility and applicability of the proposed method.

## Introduction

Genetic association analysis relies on linkage disequilibrium (LD) between alleles at two tightly linked loci [1]. With the availability of high-density maps of single nucleotide polymorphisms (SNPs), association studies have become popular tools for identifying genes underlying complex human traits and diseases [2]. It is now practical to perform genome-wide association studies (GWAS) with hundreds of thousands of SNPs in samples containing large numbers of individuals.

A common design for association studies is population-based, where unrelated subjects are collected and examined for the association between genetic variants and traits. Population-based studies are popular due to the relative ease in recruiting unrelated subjects. However, when samples are of different ethnic ancestries, population-based association studies may produce spurious associations due to population stratification, resulting in excess false positive or negative rates [3], [4]. Several methods have been proposed to deal with population stratification [5]–[11].

An alternative design uses family-based studies, where family members are collected for association analyses [12]. The application of transmission disequilibrium tests (TDT) [13], and its various extensions to a variety of genetic models for both quantitative [14]–[19] and qualitative traits [20]–[24], form the basis of family-based association tests. In these tests, the association between phenotypic traits and transmission of alleles from parents to offspring is of primary interest. TDT-based methods possess an intrinsic property of protecting against population stratification, even when only one marker is examined. However, compared with population-based samples, recruiting family members tends to be more time consuming and costly.

For most current population- and family-based GWAS, statistical power is usually limited due to the complex interplay among factors that influence the etiology of diseases [25]. A variety of approaches, e.g., increasing sample size, population selection on the degree of LD, and selecting informative tagSNPs, can improve the power for detecting association. Sample size is often restricted due to genotyping costs and limited sample resources. However, a large sample size is required to ensure sufficient statistical power to detect genes contributing subtle to moderate effects to phenotypic traits. Several recent studies that have combined unrelated subjects and nuclear families to form an enlarged sample [26]–[31] have demonstrated that analyzing combined samples can be more powerful than analyzing individual samples separately.

The problem of population stratification can arise again when analyzing combined samples, however, since neither the aforementioned correction methods for unrelated sample nor the TDT-based methods for families can be naively applied to the combined data. Thus, previous studies require a preliminary step to test whether samples from different studies can be combined. When samples are from different ethnic groups they typically fail this test [26]–[29], so an obvious limitation for these methods is that they cannot use samples from different ethnic populations. To circumvent this limitation, Zhu et al. [30] proposed to correct for population stratification in the combined sample by using principal coordinate analysis (PCoA) [8], [30], [32]. PCoA calculates principal components on individuals, and retrieves information equal to that retrieved by PCA [33]. However, when large numbers of markers (e.g. GWAS data) are involved, the calculation of PCoA by ordinary singular value decomposition (SVD) algorithms can be quite demanding in terms of both computation and computer memory. Recent work on fast matrix approximation may help speed up these calculations and save memory capacities [34], [35]. We recently proposed an extension of the method of Price et al. [6] to include familial data [36]. Compared to the method of Zhu et al. [30], this extended method can be applied to large data sets without additional demand for computation costs and computer memory.

In addition to combining samples, another approach to increasing association test power is to perform joint analysis of multiple correlated phenotypes. For many common multifactorial traits, several correlated phenotypes are usually recorded for each individual during sample collection. Joint analysis of these correlated phenotypes can theoretically provide greater power than that provided by analysis of individual phenotypes [37]. Multivariate analysis can also improve the ability to detect susceptible genetic variants whose effects are too small to be detected in univariate analysis [38], and the literature contains multiple applications of this approach to linkage studies [37]–[42]. For genetic association studies, methods have also been proposed for performing multivariate association tests on unrelated samples [43] and on families [44], separately. However, studies using multivariate analysis on combined samples are rare and further investigations using this approach are warranted [31].

In this study, we propose to perform univariate/multivariate association analysis for a combined sample of nuclear families and unrelated subjects. By use of generalized estimating equations (GEEs) [45]–[47], the proposed method assumes no specific distributions on phenotypes. Specifically, we adjust for population stratification for the combined sample by integrating principal component analysis and transmission disequilibrium test strategies. In addition, the proposed method accounts for the data of multiple siblings as well as missing parents. We evaluate the statistical properties of the proposed test through simulation studies, and demonstrate its efficacy by applying it to genetic analysis workshop 16 (GAW16) data sets.

## Results

In this section, we will evaluate the performance of the proposed method under a variety of situations by simulation. We include two methods, EIGENSTRAT [6] and FBAT [48], for comparison. EIGENSTRAT implements the method of Price et al. [6], and performs univariate association tests (continuous or binary) for unrelated samples. FBAT implements the method of Laird et al. [48] and performs family-based association tests. EIGENSTRAT and FBAT are typically used in population- and family-based association analyses, respectively, when protecting against population stratification. To make results from separate data comparable to that from the combined data, we perform the fisher product test to combine p-values from EIGENSTRAT and FBAT together to form a uniform p-value. While FBAT can perform univariate or multivariate association tests, EIGENSTRAT can only perform univariate tests. Thus, we only report the fisher product test [49] for univariate tests. We notice that a similar method of Zhu et al. [30] can also perform association tests for binary traits in combined samples. However, their current implementation requires that all nuclear families have the same structure, (e.g., the same number of offspring), a significant limitation which prevented comparison of their method to ours in simulation studies.

### Type I Error Rates

Table 1 lists type I error rates of the various tests when unrelated individuals and nuclear families are sampled. We also present results of the proposed test when analyzing unrelated samples and nuclear families separately. It is shown that the proposed test has correct type 1 error rates in all population structures when performing both univariate and bivariate analyses. Its application to unrelated samples and nuclear families, separately, also demonstrates correct error rates. The error rates for EIGENSTRAT and FBAT are also close to target levels regardless of the presence of population stratification. Thus, all tests considered here can correct for population stratification in both univariate and bivariate analyses.

**Table 1:** Type I Error Rates for Unrelated Samples and Nuclear Families.

	Nominal Level											
	5%					1%						
Population	T	T_U_	T_F_	ESTRAT	FBAT	Fisher	T	T_U_	T_F_	ESTRAT	FBAT	Fisher
One Binary Trait												
Homogeneous	4.8	5.4	5.1	5.3	3.8	4.7	0.8	1.3	0.7	1.2	0.5	0.4
Stratified	4.8	4.7	4.4	4.4	5.2	4.6	0.7	1.1	0.7	0.8	1.2	1.0
Admixture	4.9	6.1	3.1	5.9	4.3	5.7	1.2	1.2	0.8	1.5	0.2	1.1
One Continuous Trait												
Homogeneous	5.9	5.3	4.9	5.3	4.1	4.8	0.9	1.0	1.3	1.0	0.3	1.0
Stratified	6.9	6.3	5.7	5.8	5.1	4.6	1.6	0.8	0.9	0.8	1.4	0.7
Admixture	4.3	4.9	4.7	4.9	5.5	4.9	0.8	0.4	0.8	0.4	1.2	1.1
One Binary Trait and One Continuous Trait												
Homogeneous	4.6	6.8	5.4	-^a^	3.1	-	0.9	1.1	1.1	-	0.9	-
Stratified	6.0	5.5	4.7	-	5.4	-	1.3	0.9	1.1	-	1.0	-
Admixture	4.7	5.7	3.9	-	4.1	-	1.2	0.4	0.4	-	0.8	-
Two Continuous Traits												
Homogeneous	5.5	6.9	3.9	-	4.6	-	1.3	1.1	0.9	-	0.9	-
Stratified	4.7	6.0	5.1	-	5.0	-	0.9	0.9	0.9	-	1.1	-
Admixture	5.3	4.8	4.8	-	5.4	-	1.0	1.0	1.1	-	0.6	-

Notes: In homogeneous and admixture population settings, we sampled 400 unrelated subjects and 200 nuclear families when the binary trait was not involved, and sampled 200 unrelated cases, 200 controls, and 200 nuclear families with at least one affected child when the binary trait was involved. In stratified population settings, we sampled 250 unrelated subjects and 150 nuclear families from population A, and 150 unrelated subjects and 50 nuclear families from population B when the binary trait was not involved. When the binary trait was involved, we sampled 150 cases, 100 controls and 150 nuclear families with at least one affected child from population A, and 50 cases, 100 controls and 50 nuclear families from population B. Type I error rates for univariate and bivariate analyses are estimated for the combined data of unrelated samples and nuclear families under homogeneous, stratified, and admixed populations. The error rates are estimated on 1,000 replicates.

a: “-“, for EIGENSTRAT, only univariate analyses are available.

Abbreviations: T, the proposed test applied to the combined sample; T_U_, the proposed test applied to unrelated sample only; T_F_, the proposed test applied to nuclear families only; ESTRAT, the method proposed by Price et al. [6] and implemented in the software EIGENSTRAT, applied to unrelated sample only; FBAT, the program FBAT [48]; Fisher, the fisher product test on the outputs from EIGENSTRAT and FBAT.

Table 2 lists type I error rates of the various methods when unrelated individuals and sib pairs are considered. All the tests again have correct error rates that are close to target levels. Thus, the proposed test is also robust to population stratification in applications with missing parental information.

**Table 2:** Type I Error Rates for Unrelated Samples and Sib Pairs.

	Nominal Level											
	5%						1%					
Population	T	T_U_	T_F_	ESTRAT	FBAT	Fisher	T	T_U_	T_F_	ESTRAT	FBAT	Fisher
One Binary Trait												
Homogeneous	4.9	5.0	5.3	4.6	5.2	4.3	1.4	1.2	0.8	1.2	0.8	0.9
Stratified	5.7	5.7	5.5	4.9	5.5	4.5	1.2	1.1	0.7	0.9	0.8	1.0
Admixture	4.2	4.9	4.9	4.6	4.9	4.7	1.4	0.6	1.2	0.7	1.0	1.1
One Continuous Trait												
Homogeneous	4.1	4.9	4.7	4.6	4.7	5.7	0.7	1.1	0.7	1.1	0.7	1.3
Stratified	4.3	4.5	5.5	4.2	5.5	5.0	0.9	1.3	1.6	1.3	1.7	0.7
Admixture	4.6	5.6	4.1	5.3	4.2	4.8	0.8	1.7	1.0	1.7	1.0	0.9
One Binary Trait and One Continuous Trait												
Homogeneous	5.2	4.7	4.6	-	4.5	-	0.8	1.1	0.8	-	0.8	-
Stratified	4.7	5.3	5.9	-	5.6	-	1.0	1.0	1.2	-	1.2	-
Admixture	4.1	4.2	4.4	-	4.4	-	1.4	1.7	1.0	-	1.0	-
Two Continuous Traits												
Homogeneous	5.9	4.7	4.7	-	4.6	-	1.0	1.1	0.8	-	0.6	-
Stratified	5.0	5.7	4.8	-	4.8	-	0.8	1.0	1.4	-	1.4	-
Admixture	6.0	6.8	4.8	-	5.2	-	0.6	1.7	1.2	-	1.0	-

Notes: Sib pair data were obtained by deleting parental information from the simulations. Type I error rates are estimated on 1,000 replicates. See Notes in Table 1 for sample sizes and abbreviation detail.

### Power Estimates

Table 3 lists univariate power estimations for binary phenotypes when unrelated individuals and nuclear families are sampled. When analyzing combined data, the proposed test has the highest power in all genetic models. When analyzing unrelated samples alone, the power of the proposed test is approximately equal to that for EIGENSTRAT. When analyzing nuclear families alone, the power of the proposed test is significantly improved compared to FBAT. We note that parental information in FBAT is used to control population stratification, but does not contribute to the association statistic. On the other hand, parental information in the proposed method can be used to both control population stratification, and to test the association. The power improvement demonstrates that parental data are informative for testing the association.

**Table 3:** Univariate Power for Unrelated Samples and Nuclear Families (Binary Trait).

	Nominal Level											
	5%						1%					
Population	T	T_U_	T_F_	ESTRAT	FBAT	Fisher	T	T_U_	T_F_	ESTRAT	FBAT	Fisher
Homogeneous												
Recessive	30.7	12.8	12.0	22.2	15.4	15.4	11.7	3.7	3.6	8.5	4.5	4.1
Additive	90.6	54.5	53.5	80.0	49.0	69.4	77.0	29.6	28.8	58.1	26.5	49.3
Dominant	70.3	37.2	36.2	57.5	33.2	53.7	50.5	17.5	17.2	34.2	14.3	29.7
Stratified												
Recessive	55.5	31.1	29.3	41.1	25.9	37.8	31.7	13.0	11.8	21.0	9.5	19.0
Additive	92.9	60.7	59.9	80.8	54.4	78.5	81.9	36.2	36.2	62.8	27.8	53.9
Dominant	70.9	33.6	32.8	53.5	31.8	47.2	44.8	14.2	13.0	28.8	14.2	24.6
Admixture												
Recessive	59.1	23.8	24.2	49.6	28.6	38.3	36.9	10.7	9.5	25.8	10.7	16.7
Additive	94.9	67.9	67.9	91.0	70.5	79.0	85.9	32.1	33.3	67.9	38.5	57.8
Dominant	72.8	34.9	36.1	51.5	29.6	49.7	45.6	19.5	19.5	30.2	10.6	27.7

Notes: The three modes of inheritance are considered under each population structure. The causal site was assumed to render an OR value of 1.5 for homozygous mutation genotypes, heterozygous genotypes and homozygous or heterozygous mutation genotypes under recessive, additive and dominant modes of inheritance, respectively. Powers are estimated on 1,000 replicates for each setting. See Notes in Table 1 for sample sizes and abbreviation detail.

Table 4 lists univariate power estimations for continuous phenotypes. Again, the proposed test analyzing the combined data provides the highest power. Similarly, the proposed test has an approximately equal power to EIGENSTRAT when analyzing only unrelated samples, and has improved power over FBAT when analyzing only nuclear families.

**Table 4:** Univariate Power for Unrelated Samples and Nuclear Families (Continuous Trait).

	Nominal Level											
	5%						1%					
Population	T	T_U_	T_F_	ESTRAT	FBAT	Fisher	T	T_U_	T_F_	ESTRAT	FBAT	Fisher
Homogeneous												
Recessive	97.3	70.2	69.2	90.9	61.7	85.2	91.0	45.7	44.4	76.5	37.8	67.3
Additive	82.7	43.2	41.9	68.2	38.0	53.7	62.2	21.6	21.2	46.0	17.0	30.9
Dominant	92.5	54.0	53.0	80.8	47.9	67.8	80.7	29.6	29.0	60.6	25.7	47.2
Stratified												
Recessive	90.5	51.5	50.6	77.7	32.5	61.3	75.6	30.6	29.4	53.0	13.0	36.2
Additive	84.1	45.6	45.0	65.8	25.2	50.9	60.7	22.6	22.0	40.4	10.6	27.5
Dominant	94.4	61.9	61.2	84.1	43.6	72.9	84.1	37.6	37.0	66.6	20.2	48.8
Admixture												
Recessive	90.1	49.6	48.8	74.6	48.8	71.0	72.6	32.5	29.3	57.1	24.2	48.1
Additive	79.5	38.5	38.5	71.8	26.9	53.7	62.8	25.6	25.6	42.3	11.5	29.6
Dominant	95.3	63.9	63.3	85.2	43.2	74.4	82.8	35.0	33.7	60.9	23.7	53.9

Notes: The three modes of inheritance are considered under each population structure. The causal site was assumed to explain 1.0% of total phenotypic variation under each genetic setting. Powers are estimated on 1,000 replicates for each setting. Please see Notes in Table 1 for sample sizes and abbreviation detail.

Power estimations when sib pairs, instead of nuclear families, are considered are listed in Table 5 and 6 for binary and continuous phenotypes, respectively. The results are similar to those generated previously with nuclear families. Note that when analyzing only the data of sib pairs, the proposed test has similar power to FBAT.

**Table 5:** Univariate Power for Unrelated Samples and Sib Pairs (Binary Trait).

	Nominal Level											
	5%						1%					
Population	T	T_U_	T_F_	ESTRAT	FBAT	Fisher	T	T_U_	T_F_	ESTRAT	FBAT	Fisher
Homogeneous												
Recessive	20.4	14.5	14.2	11.5	11.5	14.5	7.7	4.7	4.5	3.4	3.1	5.2
Additive	74.2	55.0	53.8	36.8	36.0	63.0	51.4	30.1	28.4	16.3	16.3	37.4
Dominant	55.2	39.3	38.3	25.2	25.3	44.9	31.1	18.8	17.4	10.8	10.9	24.4
Stratified												
Recessive	49.4	33.2	31.6	20.1	20.1	29.5	24.0	14.4	13.4	6.9	7.4	14.0
Additive	82.5	62.4	61.2	42.2	42.7	70.3	58.4	37.5	35.2	17.6	17.1	47.0
Dominant	51.3	34.5	35.0	24.5	24.9	40.9	27.8	17.7	17.0	9.0	9.1	20.7
Admixture												
Recessive	36.5	25.4	24.9	16.0	16.6	35.5	18.8	9.4	8.3	6.6	7.2	13.1
Additive	81.8	56.8	57.7	40.9	40.0	71.5	60.0	36.4	34.5	21.4	21.4	44.2
Dominant	51.1	34.3	36.5	22.6	22.6	38.4	27.0	15.3	14.6	10.2	10.2	18.0

Notes: The three modes of inheritance are considered under each population structure. Sib pair data were obtained by deleting parental information from simulations. The causal site was assumed to render an OR value 1.5 for homozygous mutation genotypes, heterozygous genotypes and homozygous or heterozygous mutation genotypes under recessive, additive and dominant modes of inheritance, respectively. Powers are estimated on 1,000 replicates for each setting. See Notes in Table 1 for sample sizes and abbreviation detail.

**Table 6:** Univariate Power for Unrelated Samples and Sib Pairs (Continuous Trait).

	Nominal Level											
	5%						1%					
Population	T	T_U_	T_F_	ESTRAT	FBAT	Fisher	T	T_U_	T_F_	ESTRAT	FBAT	Fisher
Homogeneous												
Recessive	86.9	68.4	67.9	47.8	48.0	79.5	68.2	44.7	43.6	25.4	25.3	59.1
Additive	61.4	42.3	41.5	31.7	32.0	51.7	36.2	20.9	19.8	11.0	11.3	27.3
Dominant	74.4	54.5	53.8	38.4	38.6	66.8	50.6	31.1	29.5	18.5	18.7	42.3
Stratified												
Recessive	70.1	52.5	51.9	33.6	32.9	61.6	47.1	29.2	27.9	14.9	14.5	36.7
Additive	60.4	44.9	43.6	27.0	26.2	48.2	36.9	23.7	22.3	10.3	9.3	26.1
Dominant	79.3	64.5	62.8	37.1	37.0	72.5	58.6	38.0	37.0	17.0	16.7	46.5
Admixture												
Recessive	70.7	53.6	51.4	39.2	37.6	60.5	48.6	29.8	29.3	16.6	17.7	36.6
Additive	58.2	41.8	40.9	30.9	30.9	47.5	34.1	21.8	20.5	8.6	9.0	26.2
Dominant	88.3	65.7	61.3	40.9	40.9	72.0	62.8	35.0	35.8	21.2	21.2	47.1

Notes: The three modes of inheritance are considered under each population structure. Sib pair data were obtained by deleting parental information from simulations. The causal site was assumed to explain 1.0% of total phenotypic variation under each genetic setting. Powers are estimated on 1,000 replicates for each setting. Please see Notes in Table 1 for sample sizes and abbreviation detail.

Table 7 lists the gain of power by bivariate analysis for a binary trait and a continuous trait. Obviously, power for bivariate analysis is higher than both univariate analyses under all population structures. The analyses on two continuous phenotypes have similar patterns, as listed in Table 8.

**Table 7:** Power of Bivariate vs. Univariate Analyses for the Combined Data of Unrelated Samples and Nuclear Families (One Binary Trait and One Continuous Trait).

	Locus Effects	Nominal Level					
		5%			1%		
Population		T_12_	T_1_	T_2_	T_12_	T_1_	T_2_
Homogeneous							
	1.2∶0.0025	41.5	30.6	27.2	21.5	13.6	10.8
	1.3∶0.005	73.7	54.3	52.6	50.1	30.5	29.2
	1.4∶0.0075	92.3	77.7	70.5	79.2	54.5	46.5
	1.5∶0.01	98.6	90.6	82.7	93.1	77.0	62.2
Stratified							
	1.2∶0.0025	44.7	32.3	30.2	23.3	14.8	14.0
	1.3∶0.005	76.6	62.7	52.8	55.9	37.1	30.8
	1.4∶0.0075	92.7	80.4	70.9	80.8	60.8	46.7
	1.5∶0.01	98.6	92.9	84.1	93.6	81.9	60.7
Admixture							
	1.2∶0.0025	49.6	40.2	28.5	24.4	18.8	12.3
	1.3∶0.005	79.1	63.6	57.0	56.7	39.3	31.5
	1.4∶0.0075	95.0	82.5	72.5	81.9	60.6	44.3
	1.5∶0.01	100.0	94.9	79.5	94.2	85.9	62.8

Notes: Three population structures are considered. For the binary trait, the OR value ranges from 12 to 1.5. For the continuous trait, the contribution of the causal site ranges from 0.0025 to 0.01. Powers are estimated on 1,000 replicates. See notes in Table 1 for sample sizes.

Abbreviations: T_12_, the proposed test for bivariate analysis; T_1_, the proposed test for only the first trait; T_2_, the proposed test for only the second trait.

**Table 8:** Power of Bivariate vs. Univariate Analyses for the Combined Data of Unrelated Samples and Nuclear Families (Two Continuous Traits).

		Nominal Level					
		5%			1%		
Population		T_12_	T_1_	T_2_	T_12_	T_1_	T_2_
Homogeneous							
	0.0025∶0.0025	41.6	28.2	29.6	18.8	12.2	12.2
	0.005∶0.005	69.9	51.2	50.5	48.5	28.9	27.6
	0.0075∶0.0075	85.8	67.3	69.3	70.8	44.4	45.9
	0.01∶0.01	93.4	82.7	82.7	83.2	62.2	62.2
Stratified							
	0.0025∶0.0025	41.9	29.0	29.0	20.5	12.4	13.3
	0.005∶0.005	72.3	52.9	53.3	49.8	29.0	27.0
	0.0075∶0.0075	87.4	66.8	68.6	70.1	43.5	45.6
	0.01∶0.01	100.0	84.1	84.1	82.5	60.7	60.7
Admixture							
	0.0025∶0.0025	45.3	32.3	30.5	30.0	14.2	12.3
	0.005∶0.005	76.0	52.4	49.4	50.0	29.5	27.7
	0.0075∶0.0075	87.2	70.1	67.1	70.6	46.0	42.6
	0.01∶0.01	95.6	80.5	80.5	85.3	62.0	63.6

Notes: Three population structures are considered. The contributions of the causal site for both the traits range from 0.0025 to 0.01. Powers are estimated on 1,000 replicates. See notes in Table 1 for sample sizes.

Abbreviations: T_12_, the proposed test for bivariate analysis; T_1_, the proposed test for only the first trait; T_2_, the proposed test for only the second trait.

**Figure 1:**
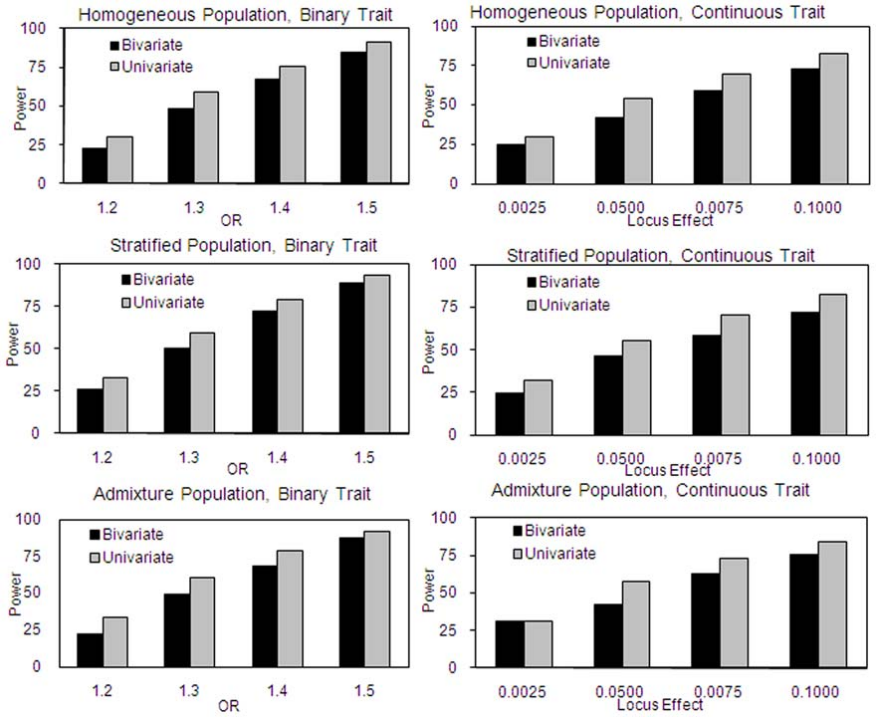
Power Estimations of Bivariate vs. Univariate Association Analysis When Genetic Variant Contributes to Only One Trait. Powers for bivariate vs. univariate analyses are estimated when the causal site contributes only to binary (left) or continuous traits (right). For binary traits, four levels of OR: 1.2, 1.3, 1.4, 1.5 with additive genetic models are considered under homogeneous, stratified, and admixture populations, respectively. For continuous traits, the causal site is assumed to explain 0.25%, 0.5%, 0.75% and 1.0% of the phenotypic variation, respectively. Powers are estimated by 1,000 replicates.

We also evaluate the loss of power of bivariate analyses in cases where pleiotropic effects are not present. Figure 1 displays the loss of power when the causal site contributes only to a binary trait (left panel) or only to a continuous trait (right panel), under the various population structures. Obviously, bivariate analysis in such cases is inferior to univariate analysis, but the power loss is relatively minor.

### Application to GAW16 Data Sets

**Figure 2:**
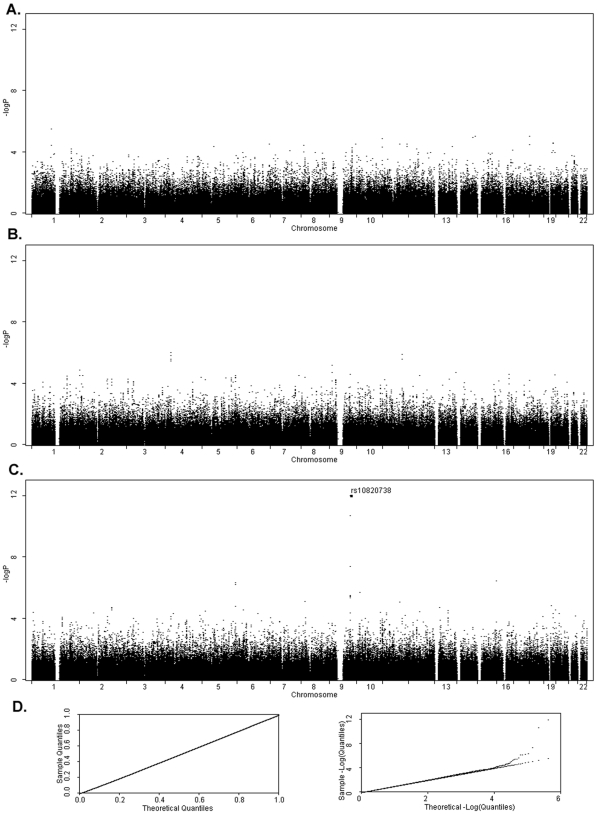
Genome-Wide Association Analyses on GAW16 Simulated HDL Data Sets. Genome-wide p-values were displayed for FBAT (A), EIGENSTRAT (B), and the proposed method (C). The marked SNP, *rs10820738*, contributes the largest effect to the trait by explaining 1.0% of phenotypic variation in the simulation. Figure 2D, quantile-quantile (QQ) plot (left) and logQQ plot (right) for the proposed method.

We apply the proposed method to GAW16 simulated data sets as described in the Methods section. Figures 2A–2C display the results of whole-genome scans by FBAT, EIGENSTRAT, and the proposed method, respectively, when analyzing the trait HDL. The most significant SNP identified by the proposed method, *rs10820738*, reaches a p-value 8.68E-13. This SNP corresponds to the major contributing gene, ABCA1, which explains 1.0% of HDL phenotypic variation in the GAW16 simulation. EIGENSTRAT and FBAT have p-values 2.30E-5 and 6.57E-4, respectively, at this SNP; neither of these methods reaches a genome-wide significant level. At the other four major genes, the proposed test also has more significant p-values than both EIGENSTRAT and FBAT, as listed in Table 9. Figure 2D (left) displays a Quantile-Quantile (QQ) plot of the proposed method. It is obvious that p-values from the proposed method distribute uniformly between 0.0 and 1.0, demonstrating the validity of the proposed method. Most of the outliers in a logQQ plot (Figure 2D, right) correspond to susceptible loci and/or their nearby SNPs.

**Table 9:** P-Values at the Major Genes for the Various Tests When Analyzing GAW16 Simulated HDL Trait.

		T	ESTRAT	FBAT
SNP	h2	P Value		
rs10820738	0.010	8.68E-13	2.30E-5	6.57E-4
rs8103444	0.002	2.53E-3	3.24E-3	0.775
rs8035006	0.003	4.60E-3	0.010	0.108
rs3200218	0.003	7.20E-6	0.014	0.011
rs8192719	0.003	3.14E-4	3.58E-4	0.508

Notes: p-values for five major genes and their contribution proportions (h2) were listed. Abbreviations: T, the proposed test; ESTRAT, the method proposed by Price et al. [6] and implemented in the software EIGENSTRAT, applied to unrelated samples only (including parents from each nuclear family); FBAT, the program FBAT [48].

We then perform bivariate analysis on the traits HDL and TG. One of the two major genes presenting pleiotropic effects, *rs3200218*, has a lower p-value (3.05E-07) in bivariate than in univariate analyses (7.20E-06 for HDL and 0.043 for TG). At the other major gene, *rs8192719*, bivariate analysis has a p-value (4.48E-04) that is approximately equal to that obtained by univariate HDL analysis (3.58E-04) (Table 10). For those loci that did not exert pleiotropic effects, however, bivariate analyses generally produce results that are of lower significance than results generated by univariate analyses.

**Table 10:** Bivariate vs. Univariate p-values at Two Major Genes Presenting Pleiotropic Effects When Analyzing GAW16 Simulated HDL and TG Traits.

	T_12_	T_1_	T_2_	FBAT_12_	FBAT_1_	FBAT_2_
SNP	P Value					
rs3200218	3.05E-07	7.20E-06	0.043	0.016	0.011	0.628
rs8192719	4.48E-04	3.14E-04	0.014	0.798	0.508	0.954

Notes: P-values for two major genes presenting pleiotropic effects to both HDL and TG were listed. Abbreviations: T_12_, T_1_ and T_2_, the proposed test applied to HDL and TG, to HDL, and to TG respectively. FBAT_12_, FBAT_1_ and FBAT_2_, the program FBAT [48] applied to bivariate analysis of HDL and TG, to univariate analysis of HDL, and to univariate analysis of TG respectively.

## Discussion

In this study, we propose a simple approach to perform univariate or multivariate association tests when correcting for population stratification with data generated by combining unrelated samples and nuclear families. Simulation studies showed that the proposed test had improved power over tests typically used to analyze family and unrelated samples separately. Further, joint analysis of bivariate traits had improved power over univariate analysis when pleiotropic effects were present. Application of the proposed method to GAW16 data sets verified its practical applicability.

By combining population- and family-based tests together, the proposed test provides flexibility in integrating technologies of family based association tests. Here, we extend the proposed model to include sib pair data with missing parental information. It is relatively straightforward to extend the proposed test to include data of general pedigrees with arbitrary structures [50]. When applied to pedigree data only, the proposed method still may have improved power over traditional TDT-based methods, as shown by analyses using the software FBAT. The power gain is attributable to the fact that the proposed method can use parental information for association tests, while the alternate methods cannot.

Combining unrelated samples and nuclear families for genetic association studies has been a focus of research for several years. Nagelkerke et al. [29] proposed using a logistic-regression model for combining case-control subjects and case-parents trios to increase statistical power. Kazeem and Farrall [28] proposed combining results of case-control tests and TDT to obtain a weighted odds ratio for a given genetic marker. Epstein et al. [27] modified the work of Nagelkerke et al. with a likelihood-based approach to allow for more flexible genetic models, such as less-restrictive assumptions of Hardy-Weinberg equilibrium (HWE) and of random mating. Chen and Lin [26] further extended the work of Epstein et al. to scenarios relaxing assumptions and estimations on mating-type distributions using a weighted least-squares approach. Jung et al. [31] recently proposed performing combined linkage and association tests for bivariate quantitative traits using a variance-component model. Despite their potential advantages, all of these methods have a requirement that both case-control subjects and case-parents come from a homogeneous population. This requirement substantially narrows the context to which these methods can be applied. The method we propose has a significant advantage in that it is robust to population stratification. Our simulation results show that the proposed test remains valid when applied to stratified or admixed populations. We note that a similar method proposed by Zhu et al. [30] can also perform association tests on combined data when correcting for population stratification. However, their current program implementation, FamCC, can only handle nuclear families with both parents available and with equal numbers of children, which rarely occurs with real data. Additionally, analyses of their method with multivariate and quantitative traits are quite limited.

Another feature of the proposed method that can improve statistical power is the ability to perform multivariate association tests. Compared with univariate models, multivariate models can be more powerful in cases where multiple traits are influenced by a common genetic variant. Notably, overall correlations between multiple traits are not necessary for multivariate analysis. In cases where the genetic variant contributes to only one trait, a loss of power will occur with the multivariate model, though the magnitude of this loss is moderate. Thus, multivariate analysis should be implemented with caution, and should only be regarded as one of the tools for detecting common susceptible loci for multiple traits.

In summary, we have developed a simple and novel method for performing univariate/multivariate association tests while correcting for population stratification, in samples combining nuclear families and unrelated subjects. The proposed method is computationally effective and can complete a typical GWAS scan within minutes. The java program implementing the proposed method, Genetic Association analysis Platform (GAP), is freely available from the authors' website (http://sites.google.com/site/zhangleira/GAP).

## Methods

We first describe our method on a combined dataset of an unrelated sample and a collection of nuclear families with both parents available. Then we extend the method to include sib pairs with missing parents, to incorporate covariates, and to correct for population stratification.

### Definitions

Assume that there are *N_f_* nuclear families and there are *n_i_* (*i* = 1, …, *N_f_*) members in the *i*th family with the first two individuals being parents. In addition, a random sample with *N_c_* unrelated individuals is also assumed available. For simplicity, we take each individual in the random sample as a separate family with size 1 so that *n_i_* = 1 for *i* = *N_f_*+1, …,, *N_f_*+*N_c_*. Thus, the total number of individuals is 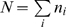, and the total number of unrelated individuals (including random sample and the two parents in each family) is *N_u_* = 2*N_f_*+*N_c_*. Assume that *K* phenotypes are available for each individual, and let **y** *_ij_* = (*y_ij_*_1_, …, *y_ijK_*) *'* be the vector of phenotypic values for the *j*th (*j* = 1, …, *n_i_*) individual in the *i*th family. Further assume that genotype data for *M* SNP markers are available for all individuals. A score *g_ijm_* at the *m*th SNP with alleles “1” and “2” is defined as 0, 1, and 2 for genotypes “11”, “12”, and “22”, respectively, for the *j*th individual in the *i*th family.

### Models

We construct our test statistic by use of previous work of score test. [17], [20], [23], [44], [48] For an individual phenotype indexed by *k*, we extend the previous work of Lunetta et al. [23] in the generalized linear model (GLM) framework, to model the association between genotype scores and phenotypes using. For a tested marker indexed by *m*, GLM relates phenotypes and genotypes by a link function (We omit the index *m* for simplicity)
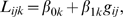
(1)where *L_ijk_* is the link function for 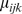, the expected value of *y_ijk_*; 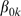 and 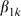 represent population mean and genotypic effect, respectively. The natural link function is the identity for continuous phenotypes, and is the logit-function for binary phenotypes.

### Defining the Score Statistic

Given genotypes, phenotypes among unrelated individuals and family members are assumed independently distributed. The log-likelihood for the sample can then be expressed as
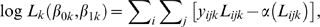
(2)where 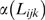 is a function of *L_ijk_* with the property 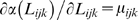, *i* = 1, …, *N_f_*+*N_c_*, *j* = 1, …, *n_i_*. The derivation of the log-likelihood with respect to 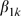 yields the score
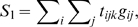
where 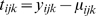. Under the null hypothesis *H*_0_ of no association (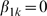), 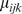 is identical to all subjects, that is, 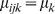.

When multiple correlated phenotypes are simultaneously modeled, it is difficult to specify the log-likelihood function (2), since joint distribution of phenotypes cannot be explicitly specified except for multivariate Gaussian distributions. For multivariate data with arbitrary distributions, Liang and Zeger [46] proposed an extension of GLM, termed generalized estimating equations (GEEs), to estimate model parameters while accounting for correlations among variables. Lange [44] further applied GEEs to genetic association analysis. Following the work of Lange, we define a multivariate score as
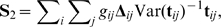
where 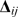 is a diagonal matrix depending on the underlying GEE model, and 
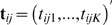
 is a vector that codes phenotypes. Under the null hypothesis *H*_0_ of no association, 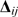 and 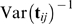 are identical to all subjects and they will vanish in the normalization of the test statistic. The resulting score under *H*_0_ is then
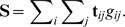



Obviously, 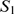 is a special case of **S** where only one phenotype is modeled.

### Distribution of the Test Statistic

The score test statistic is defined as
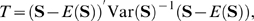
where *E*(**S**) and Var(**S**) are the mean and variance of the score, respectively. Under the null hypothesis *H*_0_, the statistic *T* will asymptotically follow a chi-square distribution with degree of freedom being the rank of Var(**S**).

For simplicity, let **Z** = **S**–*E*(**S**), so that
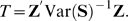

**Z** and Var(**S**) are estimated by conditioning the distribution of genotype on traits, e.g., **t** *_ij_* is fixed as constant, so that
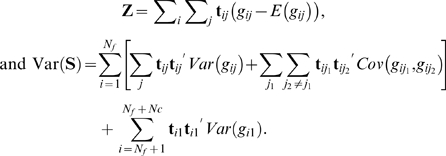



To obtain estimations for above variables, we divide the total sample into two complement sets *U* and *R*, where *U* contains *N_u_* unrelated individuals, and *R* contains the remaining *N* - *N_u_* related offspring in each family. For the set *U*, population genotype mean and variance, denoted by 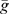 and *v*(*g*) respectively, are estimated. For each individual in the set *R*, its genotype mean and variance are estimated from its parents' genotypes according to the Mendel's law. Note that as the estimation on offspring is conditional on the parental genotypes, there will be no genotypic correlations between offspring and parents and between offspring themselves. Thus, **Z** and Var(**S**) are expressible as
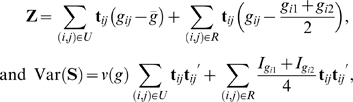
where *I_gi_*_1_ and *I_gi_*_2_ are the indicators of heterozygous genotypes for the two parents in the *i*th family.

The above expressions of **Z** and Var(**S**) render intuitive interpretations: the first part in each expression attributes to unrelated individuals, and the second part attributes to related individuals in each family. We note that when considering only related individuals in the set *R*, the second parts of **Z** and Var(**S**) constitute a family-based test statistic proposed by Rabinowitz and Laird [50] and is implemented in the software FBAT [48]. When considering only unrelated individuals in the set *U*, the first parts of **Z** and Var(**S**) constitute a valid score test in an apparent manner for random samples. Thus, the proposed test *T* can be regarded as the uniform integration of population- and family-based association tests. This characteristic allows a great deal of flexibility in including nuclear families with various structures.

### Including Data with Missing Parents

When parental genotype information is missing, the conditional means and variances for offspring genotypes in the set *R* can no longer be estimated from their parents. For families with incomplete parental data, Rabinowitz and Laird [50] propose to obtain the conditional distributions of offspring genotypes via the sufficient statistic of missing parental genotypes, which is derived from offspring genotypes and partially observed parental genotypes. By using sufficient statistic, the distributions of test statistics remain valid in the presence of population stratification. Application of the method of Rabinowitz and Laird to the proposed test with missing parents is straightforward. We replace the conditional expectations and variances of offspring genotypes in the second parts of **Z** and Var(**S**) by the ones that are estimated by conditioning on sufficient statistic of missing parental genotypes. Note that correlations among offspring in such circumstances would not vanish and they will be included in the test statistic.

### Incorporation of Covariates

When covariates are strongly predictive factors of phenotypes, incorporating them into the model can increase test efficiency. Let **W** *_ijk_* be the vector of covariates at the *k*-th phenotype for the *j*-th individual in the *i*-th family. The link function (1) modeling covariates under *H*_0_ will turn to
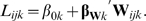



Estimation of 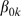 and 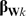 is used for construction of test statistic in follow-up steps.

We first adjust phenotypes by covariates and then construct the test statistic with the residual of phenotypes. Let **y** *_ij_* ^*^ be the residual phenotype for the *j*th individual in the *i*th family so that **t** *_ij_* = **y** *_ij_* ^*^−*μ* codes phenotypic information of subjects. The resulting test statistic *T* depends on the nuisance parameter *μ*. Though the statistic *T* remains valid regardless of the choices of *μ*, a good choice of *μ* can improve test efficiency [23]. In theory, *μ* is the population mean of phenotypes. In cases where ascertainment depends upon phenotypes, such as in case-control and case-parents designs, *μ* cannot be appropriately estimated from the sample. A variety of strategies have been proposed for different choices of *μ* to improve test efficiency [17], [20], [23], among which is the one that minimizes Var(**S**) [23]. For the *k*th phenotype, it is obvious that *Var*(*S_k_*) is the quadric form of 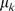, and is minimized when 
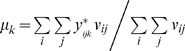
, where 
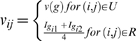
. For multivariate test, we select individual *μ* in turn for single phenotype to obtain approximate performance.

### Correcting for Population Stratification

When population stratification exists, the above test statistic may no longer be valid since the evaluations of the variance for the set of unrelated individuals are sensitive to population stratification. The adjustment for population stratification is straightforward by using our previously proposed extension [36] of PCA-based adjustment [6] that includes data with nuclear families. Briefly, we apply PCA to all unrelated individuals to calculate for each of them a vector of principal component. Individual genotypes and phenotypes are then adjusted through linear regression on principal components. For those related individuals, we propose a TDT-like strategy to infer their principal components as well as to adjust their genotypes and phenotypes. We denote the genotype score and the phenotype coding vector after adjustment as *g_ij_* ^*^ and **t** *_ij_* ^*^, respectively, for the *j*th individual in the *i*th family, and denote population genotype mean and variance after adjustment as 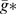 and *v*(*g* ^*^), respectively. In the appendix S1, we show that **Z** and Var(**S**) have the following forms (assuming parental information is available)
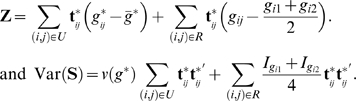



Note that the genotype deviation and variance in the second parts of **Z** and Var(**S**) are invariant to the adjustment by PCA. This is intuitively interpreted since the second parts are not affected by population stratification during construction.

### Data Simulation

To evaluate the performance of the proposed test, we conducted a variety of simulation studies. In all simulations, we simulated two SNPs with specified allele frequencies, one as causal site and the other as test site that was both tightly linked to and strongly associated with the causal site. We also simulated additional 998 random SNP markers to conduct principal component analysis, resulting in a total number of 1,000 SNPs. Both binary and continuous traits were simulated. Although the proposed test was applicable to multivariate analyses with arbitrary number of traits, we only considered bivariate situations for simplicity. For bivariate simulation, we simulated a binary trait and a continuous trait or two continuous traits. Samples were generated under one of three following population structures: a homogeneous population, two discrete populations, and an admixture population with two ancestral populations.

### Simulation 1. Homogeneous Population

In the homogeneous population structure, minor allele frequency (MAF) for both the causal and the test SNP was set to 0.2, and the allele frequency for each random SNP was drawn from a uniform distribution U(0.1, 0.9). Genotypes for unrelated individuals and parents were generated based on the corresponding allele frequencies with an assumption of linkage equilibrium between adjacent random markers, and genotypes for children in each family were generated according to parental genotypes with recombination rate 0.01 between the causal and the test sites. The number of children in each family was drawn from a Poisson distribution with mean 2.

When binary trait was not involved, we sampled 400 unrelated individuals and 200 nuclear families. When binary trait was involved, we sampled 200 cases, 200 controls and 200 nuclear families with at least one affected child. The disease prevalence was set to 30%, which was used to assign an individual's disease status under the null hypothesis. Under the alternative hypothesis, the probability of an individual being affected was calculated using the logistic regression model
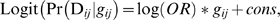
where Logit() was the logistic function; *OR* was the specified odds ratio and *cons* was a constant rendering the disease prevalence. *g_ij_* was the genotype code under recessive, additive, or dominant modes of inheritance. Unless otherwise specified, we set *OR* to be 1.5 for all simulations under alternative hypothesis.

Continuous phenotypes were drawn from normal distributions with uniform phenotypic mean and variance. Background polygenic effects were assumed to account for 40% of the phenotypic variability in simulating phenotypes for nuclear families. Under the alternative hypothesis, the causal site was assumed to explain a specified proportion of phenotypic variability under recessive, additive, or dominant modes of inheritance. Unless otherwise specified, we set the proportion explained by the causal site to 1% for all simulations under alternative hypothesis.

### Simulation 2. Two Discrete Populations

In discrete population structure, MAF at both the causal and the test site was set to 0.2 and 0.4 for two populations A and B, respectively. Allele frequencies at random markers for the two populations were generated using the Balding-Nichols model [51]. Briefly, for each marker an ancestry allele frequency *p* was drawn from the uniform distribution U(0.1, 0.9). The allele frequencies for the two populations were then drawn from a beta distribution with parameters *p*(1-*F_ST_*)/*F_ST_* and (1- *p*) (1- *F_ST_*)/*F_ST_*, where *F_ST_* was a measure of genetic distance between the two populations [52]. We set *F_ST_* to 0.05 to simulate moderate population stratification.

When binary trait was involved, we sampled 150 cases, 100 controls and 150 nuclear families from population A, and 50 cases, 100 controls and 50 nuclear families from population B. The disease prevalence in populations A and B was set to 30% and 10%, respectively, to produce the confounding effect due to population stratification. Population mean (*μ_A_* and *μ_B_*) of continuous phenotype also varied between population A and B. *μ_A_* and *μ_B_* were set such that a proportion of 20% of phenotypic variation was explained by population stratification.

Under the alternative hypothesis, phenotypes were again simulated conditional on the causal site under recessive, additive, and dominant modes of inheritance.

When the binary trait was not involved, we sampled 250 unrelated individuals and 150 nuclear families from population A, and 150 unrelated individuals and 50 nuclear families from population B. Simulation of continuous phenotypes was the same as the above.

### Simulation 3. Admixed Population with Two Ancestral Populations

In admixed population structure, we first generated two discrete populations A and B as in Simulation 2. We then adopted a continuous gene flow (CGF) model [53] to generate an admixed population from A and B. Specifically, an initial generation was produced by sampling 20,000 unrelated individuals from population A. To produce the second generation, a proportion (*λ*) of randomly selected individuals from initial population mated to individuals drawn from population B, and the remaining proportion (1–*λ*) mated among themselves. The number of children for each mating was drawn from a Poisson distribution with mean 2, and children from all marriages comprised the second generation. The second generation repeated the same process to produce the third generation, the forth, and so on. We set *λ* to 0.1 and repeated the process 5 times, resulting in the current admixed population of approximately 60%/40% of ancestry from population A/B.

We sampled the same number of unrelated individuals and nuclear families as in Simulation 1. On producing binary trait, the probability of being affected for an individual was set to 0.3*a*+0.1(1-*a*), where *a* was the ancestral proportion of population A for the individual. Similarly, phenotypic mean was set to *μ_A_ a*+*μ_B_* (1-*a*) when simulating continuous traits.

Again, under the alternative hypothesis, phenotypes were simulated conditional on genotype scores at the causal site.

Besides nuclear families, we also simulated samples of sib pairs with missing parental information, which were obtained by deleting the two parents from each family after all the above simulations.

### GAW16 Simulated Data Sets

As an application, we analyzed the Genetic Analysis Workshop 16 (GAW16) Problem 3 data sets with the proposed test. The GAW16 data sets consist of 6,476 subjects from Framingham Heart Study (FHS), where each subject has real genotypes at approximately 550,000 SNP markers and simulated phenotypes. Subjects are distributed among 3 generations and singletons. After dividing large families into smaller nuclear families and applying some quality controls to the data (for example, as the proposed test cannot analyze half-sibs, we deleted one of sibs from the data), we finally identified 5,942 subjects for analysis, 5,456 of which are family members from a total of 1,815 nuclear families and the remaining 486 are singletons. When analyzing unrelated sample, we also included parents in each family besides the 486 singletons, resulting in a total of 1,480 unrelated subjects.

A total of six correlated traits, termed HDL, LDL, TG, CHOL, CAC, and MI, respectively, are simulated on the observed genetic variation in order to mimic the lipid pathway underlying the development of cardiovascular disease [54]. Phenotype data are simulated at three pseudo-visits with 10 years apart to mimic the context of longitudinal study, and at each visit, 200 simulated data sets are replicated. We analyzed only the data set from the first replicate of the first visit, as suggested by the workshop. For univariate analysis, we focused on the trait HDL, which is influenced by five major genes each contributing 0.3% to 1% to the phenotypic variation. For bivariate analysis, we included the trait TG as well. TG is influenced by three major genes contributing to 0.3% or 0.4% to the phenotypic variation. Two major genes affecting TG also present pleiotropic effects to HDL. Both phenotypes were adjusted by age and sex.

## Supporting Information

Appendix S1 (0.03 MB DOC)
