# Supplementary material for: Univariate/Multivariate Genome-Wide Association Scans Using Data from Families and Unrelated Samples
Source: PLoS One. 2009 Aug 4;4(8):e6502. doi: 10.1371/journal.pone.0006502 (PMC2715864; doi:10.1371/journal.pone.0006502)
Supplement: Appendix S1 — (0.03 MB DOC) [file pone.0006502.s001.doc]

**Appendix**

We here show that the deviations of children’s genotype scores to expectations and genotypic variance remain the same even after adjustment by PCA. For a nuclear family with both parents available, denote the genotype score for the father and the mother as *g_f_* and *g_m_* respectively. For a particular child’s genotype, denoted as *g_c_*, the deviation to expectation and variance are

$\Delta=g_{c}-\frac{g_{f}+g_{m}}{2}$,

and $var=\frac{I_{g_{f}}+I_{g_{m}}}{4}$, where *I_gf_* and *I_gm_* are the indicators of heterozygous genotypes. The parents’ genotype scores after adjustment are assumed to be

*g*_f_^*^ = *g_f_* – *a*,

and *g_m_*^*^ = *g_m_* – *b*.

We previously proposed that the child’s genotype score after adjustment would be

$$g_{c}^{*}=g_{c}-\frac{a+b}{2}$$

Thus, the deviation after adjustment will be

$$\begin{matrix} \Delta^{*}=g_{c}^{*}-\frac{g_{_{f}}^{*}+g_{_{m}}^{*}}{2} \\ =g_{c}-\frac{a+b}{2}-\frac{g_{f}-a+g_{m}-b}{2} \\ =\Delta\end{matrix}$$

Since the genotypes scores for all possible children in the family are adjusted by the same constant (a+b)/2, the variance of genotype will not change after adjustment, that is, $var^{*}=var=\frac{I_{g_{f}}+I_{g_{m}}}{4}$
